# Post-translational protein lactylation modification in lung cancer: an emerging targeted therapeutic strategy

**DOI:** 10.3389/fimmu.2026.1734414

**Published:** 2026-03-11

**Authors:** Yongxuan Li, Zhao Li, Xingfei Liu, Zhengzhou Qiu, Ruilin Zhang, Huan Li, Chenggen Gao, Changying Guo

**Affiliations:** 1 Jiangxi Medical College, Nanchang University, Nanchang, Jiangxi, China; 2 Department of Thoracic Surgery, Jiangxi Cancer Hospital, The Second Affiliated Hospital of Nanchang Medical College, Jiangxi Cancer Institute, Nanchang, Jiangxi, China; 3 Department of Cardiothoracic Surgery, Suzhou BOE Hospital, Suzhou, Jiangsu, China; 4 Department of Oncology, The Forth Affiliated Hospital of Soochow University, Suzhou, Jiangsu, China; 5 Suzhou Medical College, Soochow University, Suzhou, Jiangsu, China

**Keywords:** immune evasion, lactylation, lung cancer, metastasis, targeted therapy, therapeutic resistance

## Abstract

Lung cancer remains the second most prevalent malignancy worldwide and is characterized by persistently high incidence and mortality rates. As the disease progresses, most patients develop immune evasion and metastatic dissemination, which represent major threats to overall survival. The advent of targeted therapies and immunotherapies has fundamentally reshaped the clinical management of lung cancer; however, therapeutic resistance and limited durability of response remain critical challenges. Lactylation has recently emerged not only as a novel post-translational modification but also as a potential therapeutic vulnerability in lung cancer. By modulating the activity, stability, and transcriptional functions of both histone and non-histone proteins, lactylation reshapes tumor metabolism, immune evasion, and resistance-associated signaling pathways. Importantly, growing evidence suggests that therapeutic strategies targeting lactylation-related pathways may offer new opportunities to improve outcomes in patients with advanced lung cancer and overcome acquired resistance to existing therapies. In this review, we systematically delineate the molecular mechanisms underlying lactylation, with particular emphasis on the enzymatic machinery governing lactylation dynamics and its regulatory network. We synthesize current evidence describing how lactylation-driven signaling programs contribute to lung cancer progression, immune escape, and treatment resistance, highlighting the complex interplay between lactylation pathways and lung cancer pathobiology. Furthermore, we critically evaluate the translational potential of lactylation sites and their downstream effectors as diagnostic and prognostic biomarkers, as well as actionable therapeutic targets. Collectively, these findings support the concept that targeting lactylation-associated regulatory circuits represents an emerging and potentially more selective therapeutic strategy for lung cancer. Nevertheless, clinical translation remains constrained by the lack of specific intervention tools, standardized detection methodologies, and robust human data. Future studies should prioritize the development of precise lactylation-targeted approaches and large-scale, longitudinal clinical investigations to validate their clinical value and overcome current translational bottlenecks.

## 1 Introduction

Epidemiological data indicate that lung cancer represents 12.4% of the global cancer incidence and approximately 18.7% of total cancer-related deaths annually (1, 2). The high prevalence and mortality of lung cancer constitute a significant public health challenge, primarily because of late-stage diagnosis, pronounced metastatic potential, and limited therapeutic interventions. In recent years, the Warburg effect—a hallmark of metabolic reprogramming—has been demonstrated to directly contribute to lung cancer progression by altering tumor cell metabolism (3). Lactate, the primary metabolite of this process, has been shown to regulate gene transcription at the epigenetic level through a post-translational modification (PTM) termed lactylation. This modification occurs when lactate concentrations reach a threshold, enabling the covalent binding of lactate groups (la) to protein lysine residues (4). As a novel epigenetic modification, lactylation differs fundamentally from classical genetics in that it enables heritable changes in gene expression without altering the DNA sequence. Studies have revealed that lactylation is widespread in human lungs. Under normal physiological conditions, 451 lactylated proteins and 724 lactylation sites have been identified (5). While some of these modifications serve regulatory functions in homeostasis, excessive lactate accumulation can trigger a surge in both histone and non-histone protein lactylation. Lactylated histones regulate the accessibility of transcription factors and epigenetic regulators to genetic material by modulating chromatin compaction, thereby influencing gene expression and tumorigenesis (6). Moreover, the lactylation of non-histone proteins remodels the tumor microenvironment (TME) by modulating tumor metabolism. These lactylation-driven alterations in genes and their associated pathways play critical roles in lung cancer progression and are frequently correlated with adverse prognostic factors, including immune evasion, invasion, metastasis, and acquired resistance (7, 8).

Despite significant improvements in the survival rates and quality of life of lung cancer patients following the advent of targeted drugs and immune checkpoint inhibitors (ICIs) (9), acquired resistance remains the primary limitation to treatment efficacy. Therefore, strategies aimed at restoring immune activity and reversing tumor resistance are highly important for improving clinical outcomes. Given the complex and intimate relationship between lactylation and lung carcinoma, a growing number of therapeutic agents targeting the lactylation process and specific lactylation sites are emerging. These findings highlight the potential of lactylation as a novel therapeutic target and a promising diagnostic and prognostic biomarker. Establishing a comprehensive framework of lactylation mechanisms is critical for elucidating lung cancer pathogenesis and expediting the exploration of novel therapeutic strategies.

## 2 The lactylated blueprint of lung cancer: mechanisms that matter

### 2.1 The accumulation and transport of lactate constitutes the basis of lactylation

As a respiratory and metabolic organ, the lung possesses the dual capacity to produce and release lactate. Its unique anatomical structure, which is in direct contact with the external environment, makes it the primary target organ for environmental carcinogens such as Particulate Matter (PM2.5), formaldehyde, and radon. This characteristic further exacerbates aberrant remodeling of lactate metabolism in lung cancer, rendering it one of the core hallmarks of metabolic reprogramming in this disease. Compared with other solid tumors, lactate metabolism in lung cancer exhibits four distinctive features.

First, the exogenous exposure–glycolysis activation axis. Lung tissue is directly exposed to the external environment via the airways, and carcinogens such as PM2.5 and radon can disrupt aerobic oxidation and drive enhanced glycolysis through specific mechanisms, thereby serving as “exogenous inducers” of lactate production. Specifically, PM2.5 induces the expression of dihydrolipoamide S-acetyltransferase (DLAT) to promote glycolytic reprogramming, whereas radon exposure damages Tumor Protein p53-dependent mitochondrial function–related signaling cascades, forcing metabolic flux toward the glycolytic pathway. Together, these processes promote lactate generation and malignant cellular transformation (10, 11).

Second, lung cancer exhibits a distinctive high-lactate phenotype against the paradox of oxygen partial pressure. Despite lung tissue being exposed to oxygen and having an oxygen partial pressure several times higher than that of other organs (12, 13) —a condition that would typically favor Oxidative phosphorylation—lung cancer tissues still exhibit elevated glycolytic rates and rank among the top lactate producers across solid tumors. In A549 cells, the glucose uptake rate is tenfold that of glutamine (14), a metabolic feature standing in sharp contrast to pancreatic cancer, where glutamine-dominated circular metabolism prevails. This highlights that lactate acts as the major contributor to the circulating metabolism of lung cancer (15), a finding further supported by the observation that lung-homing tumor cells display the highest levels of glucose uptake and lactate production (16).

Third, multifactorial cooperation promotes lactate accumulation. This process is closely linked to hypoxia and abnormal vascular structure and function arising from rapid tumor cell proliferation. Approximately 85% of glucose within tumor cells is metabolized to pyruvate via glycolysis, which is then converted to lactate, while poorly vascularized regions exhibit inefficient lactate clearance (17, 18). Meanwhile, high-frequency mutations in KRAS (Kirsten rat sarcoma viral oncogene) and EGFR (epidermal growth factor receptor)—although not lung cancer-specific—are uniquely prevalent in lung cancer and enhance glycolysis through multiple signaling pathways to promote metabolic reprogramming (19–21). Beyond the intrinsic metabolic activity of tumor cells, intratumoral microbiota also contributes to lactate accumulation to a certain extent. The traditional notion of “sterile tumors” has been overturned, and intratumoral microbiota have emerged as a cutting-edge research focus in oncology in recent years (22). Studies have demonstrated that the lung cancer-associated microbiota exhibit significant differences from those in healthy lung tissue and can directly modulate the biological behavior of tumor cells. Among them, resident intratumoral microbes such as Staphylococcus and Lactobacillus possess lactate-producing capacity and have been shown to supply lactate to lung cancer cells and the TME (23, 24). However, the low microbial biomass in the lung results in a relatively small amount of lactate produced by intratumoral microbes. Nonetheless, microbe-derived lactate still engages in intricate crosstalk with lung cancer cells to drive lung cancer progression (25).

Furthermore, Glutaminolysis, a hallmark of cancer metabolism, also serves as an important source of lactate production. Tumor cells exhibit a preference for aerobic glycolysis, which leads to the accumulation of large amounts of glycolytic intermediates that are shunted toward biosynthetic processes. This results in insufficient pyruvate to sustain mitochondrial function, whereas glutaminolysis replenishes tricarboxylic acid (TCA) cycle metabolites (e.g., α-ketoglutarate and succinate) through anaplerotic reactions, thereby maintaining the biomass of tumor cells (26). In this process, malate, a downstream product of α-ketoglutarate in the TCA cycle, is exported from mitochondria to the cytoplasm, decarboxylated to pyruvate by malic enzyme, and subsequently converted to lactate under the catalysis of lactate dehydrogenase (LDH). Meanwhile, glutamate generated from glutaminolysis is catalyzed by aspartate aminotransferase to form aspartate; after being transported to the cytoplasm, aspartate undergoes a reverse transamination reaction to regenerate oxaloacetate, which is ultimately converted to lactate (27).

In addition, under hypoxic conditions in lung cancer, hypoxia-inducible factors (HIFs), which drive a range of biological effects, are regulated by cytokines such as nuclear factor of activated T-cells cytoplasmic 2 (NFATc2) (28), further elevating lactate levels. Lactate, in turn, acts as a regulator of intercellular communication and induces the positive feedback activation of HIFs; this subsequently promotes the transcription of genes including lactate dehydrogenase A (LDHA) and pyruvate dehydrogenase kinase 1 (PDK1), thereby forming a positive feedback loop of lactate production that leads to massive lactate accumulation (29).

Fourth, enhanced transport capacity mediated by high monocarboxylate transporters (MCTs) expression. Accumulated lactate is shuttled bidirectionally within and between cells, tissues, and organs via MCTs (30). MCT1 expression is upregulated under high lactate concentrations, whereas MCT4 expression is increased when lactate levels are low (31). This dynamic regulation endows lactate with signaling molecule functions (32), laying the foundation for lactylation in lung cancer. Although MCT expression has been widely characterized in cancer cell lines and various tumor types, strikingly high expression is observed predominantly in lung cancer (33). This regulatory pattern not only prevents intracellular acidosis caused by excessive lactate accumulation but also maximizes the signaling functions of lactate, representing one of the key reasons why lactylation levels in lung cancer are higher than those in other tumors.

However, significant controversies and gaps remain to be addressed in current research on lung cancer metabolism. First, a unified framework for defining metabolic profiles across different pathological types, molecular subtypes, and clinical stages of lung cancer has yet to be established, resulting in fragmented and sometimes inconsistent conclusions regarding the association between metabolic phenotypes and tumor classification. Second, the relative contributions of tumor cell–intrinsic metabolic pathways (e.g., enhanced aerobic glycolysis and glutaminolysis) and such microenvironment-driven adaptive metabolic alterations (e.g., metabolic reprogramming of cancer-associated cells and microbial crosstalk) have not yet been precisely delineated (17, 23, 27, 34), which substantially limits the clinical translational value of related research findings.

### 2.2 Two pathways for the occurrence of lactylation

The human body contains two lactate isomers. Under normal conditions, the vast majority of lactate produced is the L-isomer, whereas a minor D-isomer is generated via the glyoxalase system (35, 36). These two enantiomers give rise to distinct pathways of lactylation. The pathway involving L-lactate is termed enzyme-dependent lactylation, as it requires the coordinated action of “writer” enzymes (lactyltransferases), “eraser” enzymes (delactylases), and “reader” proteins that recognize lactylated lysines. In contrast, D-lactate participates in a non-enzymatic lactylation process that occurs through an irreversible intramolecular SN transfer reaction (37). In addition, another lysine lactylation (Kla) isomer in the non-enzymatic pathway, termed Kce, can be directly generated from methylglyoxal (MGO), an upstream metabolite of D-lactate (38). Although non-enzymatic lactylation isomers have been reported for a long time, the biological functions of most of them remain poorly characterized, particularly with regard to their roles in the initiation and progression of lung cancer.

### 2.3 Key enzymes mediating dynamic regulation of protein lactylation in lung cancer

During investigations into the mechanisms underlying lactylation, lactyltransferases and delactylases that catalyze the dynamic equilibrium of lactylation have emerged as central research targets, and their dysregulation is closely associated with metabolic reprogramming and malignant progression in lung cancer. On the basis of the high mechanistic similarities between lactylation and acetylation, Zhang et al. replaced acetyl-CoA with lactyl-CoA and were the first to demonstrate the ability of E1A-binding protein p300 (p300) to function as a lactyltransferase (39, 40). Moreover, this activity can regulate histone H3K18 modification in non–small cell lung cancer (NSCLC) cells, thereby promoting lung cancer cell proliferation. This finding reveals the dual “acetyl–lactyl transferase” functional properties of the lysine acetyltransferase (KAT) family in lung cancer and lays a foundation for subsequent studies. With the deepening of research, additional lung cancer–related lactyltransferases have been progressively identified. HBO1, a member of the MYST subfamily of the KAT family, is transcriptionally upregulated in NSCLC. It not only regulates chromatin remodeling through acetylation (41), but has also been shown to mediate H3K9 lactylation, thereby promoting oncogene expression and lung cancer cell growth (42). Notably, recent studies have challenged the traditional “lactyl-CoA–dependent” model of lactylation, demonstrating that the aminoacyl-tRNA synthetase family members alanyl-tRNA synthetase 1/2 (AARS1/2) function in lung cancer as both L-lactate sensors and lysine lactyltransferases. They can directly utilize L-lactate, which binds to ATP and undergoes a two-step reaction to complete the lactylation process (43, 44). This provides a novel molecular mechanism for enzyme-dependent lactylation. In addition, although another member of the same family, GCN5 (general control non-derepressible 5, KAT2A), has been shown to activate the expression of repair genes by mediating H3K18 lactylation in monocytes after myocardial infarction, and is highly expressed in lung adenocarcinoma (LUAD) and involved in methylation-mediated regulation of tumorigenesis (45, 46), whether it possesses lung cancer–specific lactyltransferase activity and whether it functionally cooperates with p300/HBO1 remain unclear. Direct experimental evidence is still lacking, warranting further investigation.

Lactylation is a dynamically regulated epigenetic modification. Delactylases in lung cancer are also primarily derived from the cross-functional activities of the histone deacetylase (HDAC) family, which play fundamental roles in epigenetic regulation of gene expression and chromatin remodeling. Aberrant HDAC expression and dysregulated chromatin lactylation are thought to be associated with lung cancer pathogenesis and therapeutic resistance (47). However, distinct HDAC subtypes exhibit markedly different modes of action and regulatory priorities: class I HDAC members HDAC1 and HDAC3, as well as SIRT2 of the class III HDAC (sirtuin) family, are aberrantly overexpressed in lung cancer (48–50). They operate beyond the canonical acylation regulatory framework and participate in non-histone delactylation processes in lung cancer cells (44, 51, 52);

Notably, current research on delactylases in lung cancer remains subject to significant limitations. On the one hand, HDACs, particularly HDAC1 and HDAC3, have been identified as potential delactylases capable of removing lysine lactylation modifications. Paradoxically, however, histone deacetylase inhibitors (HDACi), which abrogate HDAC enzymatic activity, have demonstrated therapeutic efficacy in lung cancer (53), with belinostat and chidamide being typical examples (54, 55). This observation presents an apparent paradox: theoretically, inhibition of HDAC-mediated delactylation would elevate cellular lactylation levels, yet accumulating evidence has consistently implicated lactylation in the promotion of lung cancer progression. This notable paradox is likely attributable to the broad substrate specificity of HDACs—beyond modulating lactylation, HDACi simultaneously alter multiple acylation pathways, including acetylation and palmitoylation. Furthermore, differences in substrate affinity and context-dependent regulatory effects of HDAC1 and HDAC3 may enable the overall anti-tumor efficacy of HDACi to outweigh the potential pro-tumor risks associated with elevated lactylation levels, and the precise underlying mechanisms remain to be elucidated. On the other hand, whether lactyltransferases and delactylases form a dynamic equilibrium network, and how this network responds to lung cancer initiation and progression to influence clinical therapy, still require systematic investigation. This expanding repertoire of regulatory proteins (see **Table 1**) is essential for uncovering the pathophysiological and clinical implications of lactylation, and provides a theoretical basis for the development of histone lactylation inhibitors.

**Table 1:** Key enzymes involved in the lactylation modification process.

Type	Name	Targets
writer	CBP (CREB-binding protein, KAT3A)	ENO1(enolase 1)-K89 (60)
	P300(KAT3B)	APOC2(apolipoprotein C2)-K70 (61), H3K18 (62, 63), H4K8 (40)
	TIP60 (60 KDa Tat-Interactive Protein, KAT5)	NBS1(Nijmegen breakage syndrome 1)-K388 (64)
	HBO1(KAT7)	H3K9 (42)
	AARS1/2	SUMO2(small ubiquitin-like modifier 2)-K11la (44)
eraser	HDAC1	SUMO2-K11la (44)
	HDAC3	APOC2-K70 (61), NBS1-K388 (64), RBM(RNA-binding motif protein)-K850 (51)
	SIRT2	LDHA-Kla (52)

Reader proteins serve as critical bridges connecting lactylation modifications to cellular functional changes. However, research on lactyl-specific readers remains in its infancy. In acetylation, “readers” rely on structural domains such as bromodomains (BRDs) and double plant homeodomain finger (DPF) motifs to recognize modified lysines. Given the consistency of acylation reactions, the conservation of bromodomains, and the specificity of binding, acetyl-lysine reader proteins such as Brg1 (Brahma-related gene 1) and DPF2 have been proposed to also recognize lactyl groups and thereby regulate transcription (56, 57), a hypothesis that has been validated in human pluripotent stem cells and cervical cancer cells (58, 59).

Although lung cancer–specific reader proteins have not yet been identified, current proteomic technologies provide promising avenues for their discovery. For example, approaches such as screening predicted protein domains using *in vitro* lactylation-labeled protein probes, performing high-throughput *in vitro* screening with synthetic peptide microarrays, identifying interacting proteins co-immunoprecipitated with bait proteins by mass spectrometry, and applying genetic code expansion (GCE) technologies to site-specifically incorporate noncanonical amino acids (ncAAs) carrying photoaffinity probes into full-length proteins all show considerable potential. Nevertheless, these techniques have inherent limitations. Weak or transient interactions are difficult to detect; moreover, the short capture radius of photoaffinity probes and the potential perturbation of interactions between reader proteins and lysine lactyl groups caused by photocrosslinking moieties represent additional important constraints.

## 3 Uncovering lactylation as a key driver of lung cancer progression

### 3.1 The dual roles of histone and non-histone lactylation

As a post-translational protein modification, lactylation primarily occurs on the flexible N-terminal tails and globular core regions of core histones. The currently identified Kla sites are predominantly on histones H3 and H4, including H3K18, H3K14, H3K9, H4K8, H4K16, and H4K12. Lactylated histones, on the one hand, influence the tight binding between histones and DNA, as well as the interactions among histones, DNA, and associated factors, by altering chromatin structure, thereby disrupting the balance of gene transcription (65). On the other hand, they serve as docking sites for reader proteins that recruit additional chromatin modifiers and remodelers, facilitating bidirectional interactions (66), and through the accumulation of diverse genetic and epigenetic modifications, they enable cells to adapt to the metabolic demands associated with rapid proliferation (29).

**Figure 1:**
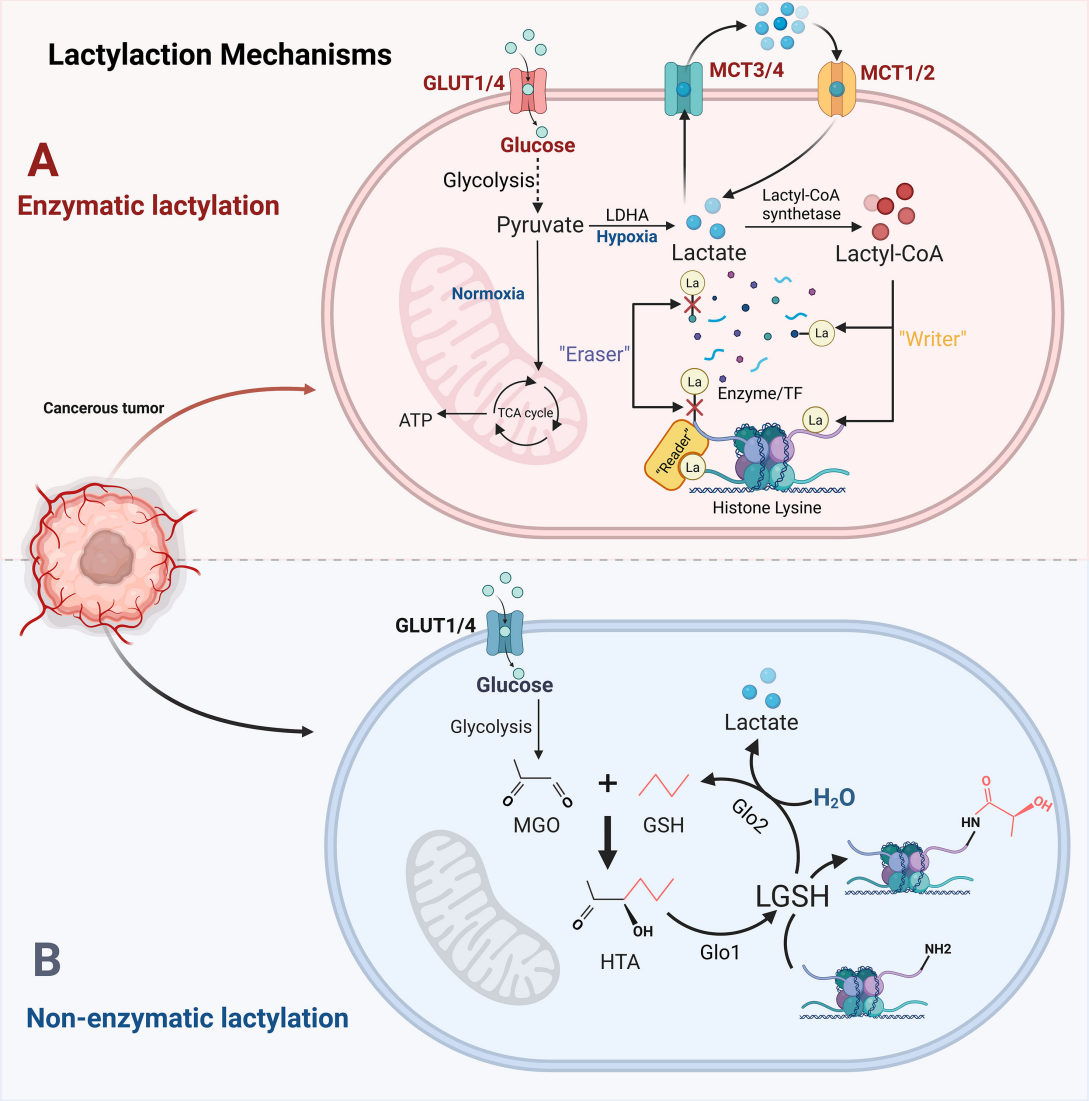
Two pathways for the occurrence of lactylation. Schematic overview of enzymatic and non-enzymatic protein lactylation in cancer cells. **(A)** Enzymatic lactylation: Glucose-derived lactate is generated through glycolysis and LDHA activity, transported by MCTs, and converted into lactyl-CoA, which serves as a donor for lysine lactylation catalyzed by putative writers and reversed by erasers, thereby regulating chromatin structure and gene transcription. **(B)** Non-enzymatic lactylation: Glycolytic by-products are processed through the glyoxalase system to form lactoylglutathione, which non-enzymatically transfers lactyl groups to lysine residues on histone and non-histone proteins. Together, these pathways link altered glucose metabolism to widespread protein lactylation in cancer cells. Created with BioRender.

In addition to histones, a range of non-histone proteins—including metabolic enzymes, transcriptional regulators, and signaling molecules—are also subject to lactylation. For example, in lung cancer, the insulin-like growth factor 1 receptor (IGF1R, involved in cell growth and survival control) can be significantly stabilized through lactylation (67). Additionally, poly (ADP-ribose) polymerase 1 (PARP1, involved in cell differentiation, proliferation, and tumor transformation) undergoes formaldehyde-induced lactylation, which enhances its activity and thereby promotes lung cancer cell growth (68). In fact, non-histone proteins themselves are more abundant than histones in lung cancer cells and participate in a broader range of biological processes. Consequently, lactylation occurring on non-histone proteins may exhibit greater abundance and specificity compared with histone lactylation (69). Thus, lactylation occurring on non-histone proteins may exert a far more profound impact on lung cancer cells. The neglect of non-histone lactylation in existing studies has directly resulted in a systemic incompleteness of lung cancer regulatory networks. As key executors of core processes such as metabolic reprogramming, DNA repair, signal transduction, and immune responses, non-histone proteins harbor the potential to serve as central hubs linking enhanced glycolysis, immune checkpoint regulation, and other processes through their Kla modifications. However, current research has largely failed to explore this potential, leading to a pronounced imbalance between functional importance and research attention. Given the widespread presence of Kla sites on non-histone proteins, future studies should prioritize the identification and characterization of additional non-histone proteins undergoing Kla modification in cancer cells. This will place higher demands on high-throughput sequencing, proteomics, and bioinformatics tools to accurately evaluate the functional consequences and interaction networks of these modified proteins (see **Figure 1**).

### 3.2 Lactylation hijacks immunity: a sweet spot for tumor escape

Under immune selective pressure, NSCLC cells and the TME undergo co-evolution to establish an immunosuppressive phenotype. Lactylation, as a key epigenetic modification linking metabolic reprogramming and immune regulation, has been shown to drive immune evasion through multidimensional and multicellular regulatory networks. Its regulatory mechanisms can be broadly categorized into two major dimensions: “tumor cell–intrinsic regulation” and “immune cell polarization within the TME,” with pronounced cooperativity and subtype specificity among different mechanisms. On the one hand, lung cancer cells promote the expression of immune checkpoint molecules through the H3K18la–MYC (MYC proto-oncogene)–POM121 (nuclear pore membrane protein 121)–PD-L1 (programmed death-ligand 1) regulatory axis, directly suppressing effector T-cell function and thereby facilitating immune evasion (8, 70). On the other hand, the cyclic guanylate (GMP)-adenosine monophosphate (AMP) synthase-stimulator of interferon genes (cGAS−STING) pathway, a key target in the immune evasion mechanism of lung cancer (71), is also regulated by lactylation. In NSCLC cells, aberrant dsDNA derived from genomic instability is detected by cGAS, a cytoplasmic DNA sensor, which then catalyzes the production of cyclic GMP−AMP (cGAMP). cGAMP binds to and activates STING located on the endoplasmic reticulum, thereby triggering innate antitumor immune responses (72). However, during tumor progression, lung cancer cells inhibit the cGAS−STING pathway through multiple mechanisms (e.g., glycolysis-dependent post-translational modifications) to evade immune surveillance (73). This phenomenon has also been observed in other cancer types. For instance, lactylation at K317 of the DNA damage repair protein KU70 significantly suppresses cGAS−STING signaling and maintains an immunosuppressive TME in glioma (74). In lung cancer, lactylation disrupts the cGAS−STING pathway via two mechanisms. First, lactylation at K21 of cGAS causes its dissociation from the antioxidant factor NQO1 (NAD(P)H quinone dehydrogenase 1), followed by recognition by the proteasome subunit PSMA4, thereby promoting its ubiquitination-independent degradation. Second, the PI3K−mTOR pathway is a major regulator of the kinase activity of ULK1 (unc-51 like autophagy activating kinase 1). Lactylation at K415 of the catalytic subunit PI3KCB of PI3K directly inhibits ULK1-mediated phosphorylation of PSMA4, which further enhances PSMA4-dependent degradation of cGAS (75, 76).

To date, studies on the regulation of the cGAS−STING pathway by lactylation have mainly focused on cGAS. Although STING has been reported to undergo lactylation induced by glycolysis-derived lactate in microglia and contribute to neuropathic pain (77), and to potentiate glycolysis and promote histone lactylation at the HK2 (Hexokinase 2) promoter by stabilizing HIF1α in macrophages, forming a feedback loop for metabolic reprogramming (78), direct evidence supporting STING lactylation in the lung cancer context remains lacking. The crosstalk between lactylation and STING in lung cancer therefore warrants further investigation.

H3K18la-driven polarization of immune cell heterogeneity within the TME represents another critical component of immune evasion in lung cancer. Specifically, H3K18la promotes the expression of cytokines and markers such as Arg1 (arginase 1, catalyzes the hydrolysis of arginine to ornithine and urea), SLC2A1 (solute carrier family 2 member 1, also known as GLUT1, which mediates cellular glucose uptake), and VEGF (vascular endothelial growth factor, promote vascular endothelial cell proliferation and angiogenesis), thereby driving macrophage polarization toward the pro-tumorigenic M2 phenotype and facilitating local immune escape (79, 80). Moreover, in a unique liquid TME—malignant pleural effusion (MPE)—H3K18la continues to exert cooperative immunosuppressive effects (81). Because MPE occurs predominantly in advanced-stage lung cancer, the relatively specific mechanisms operating within this milieu become key drivers of disease progression and immune evasion (82). On the one hand, H3K18la participates in the transcriptional regulation of nuclear factor kappa-B p65 (NF-κB p65, regulate transcription of genes associated with inflammation and immune responses), upregulating tumor necrosis factor receptor 2 (TNFR2) expression in regulatory T cells (Tregs) and thereby strengthening Treg-mediated immunosuppression of CD8^+^ T cells (83). On the other hand, H3K18la induces upregulation of C-X-C motif chemokine ligand 16 (CXCL16) expression in fibroblasts, forming a CXCL16–CXCR6 axis with TNFR2^+^ Tregs that highly express the C-X-C motif chemokine receptor 6 (CXCR6). This axis further recruits TNFR2^+^ Tregs into the MPE and promotes their immunosuppressive activity and disease progression (84). In addition, H3K18la can induce aberrant expression of FOXP3 (forkhead box P3) in NKT-like cells, driving high expression of immune checkpoint molecules such as PD-1 (programmed death 1), PD-L1, CTLA-4 (cytotoxic T-lymphocyte-associated protein 4), and LAG-3 (lymphocyte-activation gene 3), as well as immunosuppressive cytokines including TGF-β (transforming growth factor β) (81). This shift converts these cells from an antitumor phenotype to an immunosuppressive phenotype, further expanding the spectrum of cell types through which lactylation regulates immune evasion.

**Figure 2:**
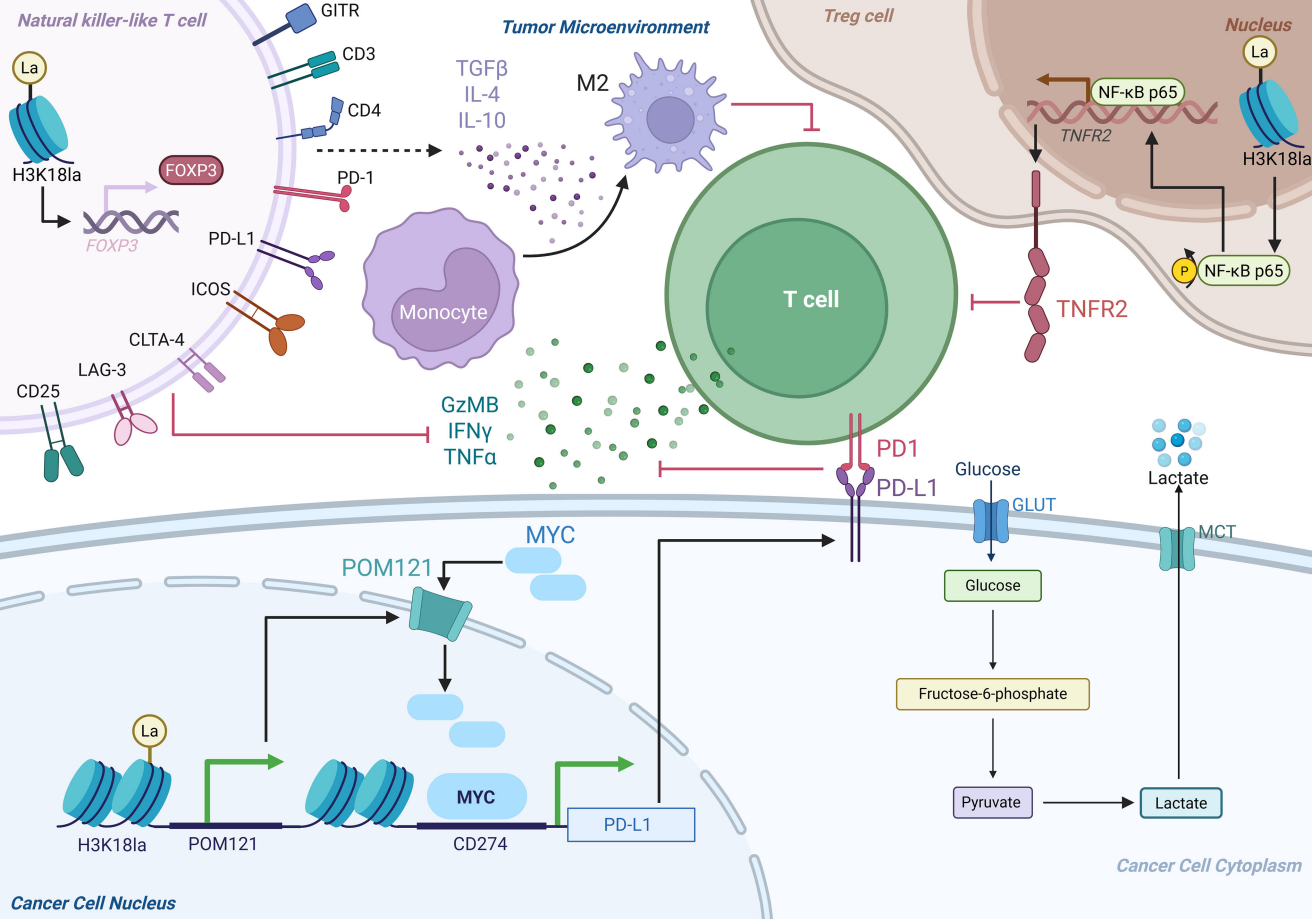
Immune escape induced by lactylation in lung cancer. Schematic illustration of how lactylation promotes tumor immune evasion. Enhanced glycolysis and lactate accumulation induce histone lactylation (e.g., H3K18la), leading to transcriptional activation of immune-regulatory genes, including MYC-dependent PD-L1 expression in tumor cells. Elevated PD-L1 suppresses cytotoxic T-cell effector functions via PD-1 signaling. Concurrently, lactylation supports regulatory T-cell stability, M2 macrophage polarization, and an immunosuppressive cytokine microenvironment, collectively fostering immune escape. Created with BioRender.

However, existing studies have largely described these mechanisms in isolation, without carefully dissecting their interrelationships. Current evidence does not demonstrate whether these mechanisms are activated simultaneously or act cooperatively, nor can it exclude the possibility of “competitive inhibition” among them under immune selective pressure (79, 80, 83, 84). Moreover, important gaps remain within these immune evasion pathways. For example, the cellular origin of Tregs recruited via the CXCL16–CXCR6 axis remains unclear, making it difficult to determine whether they arise from within the tumor itself or from the peripheral circulation (84). In addition, current technical limitations affect the precision of these studies. Experimental modulation of histone lactylation levels has primarily relied on inhibiting glycolysis (e.g., LDHA knockout) or directly altering lactate concentrations (e.g., sodium lactate supplementation). To date, no specific inhibitors or genetic tools are available to selectively target H3K18la. Although these approaches can alter H3K18la levels and downstream gene expression, they cannot specifically isolate the unique functions of H3K18la and may introduce off-target effects (see **Figure 2**) (83–85).

### 3.3 Lactylation unleashed: fueling cancer cell invasion and metastasis

Metastasis is a defining hallmark of malignant tumors, and its initiation is often triggered by epigenetic factors, such as lactylation, induced by environmental stimuli (86). At the mechanistic level, lactylation cooperatively regulates uncontrolled tumor cell proliferation, epithelial–mesenchymal transition (EMT) programs, pro-invasive intercellular signaling, and angiogenic remodeling, ultimately establishing malignant growth in distant organs (87).

First, EMT is a critical step through which lung cancer cells acquire mesenchymal phenotypes and invasive capacity. Lactylation promotes the stable establishment of mesenchymal traits by directly modifying transcription factors or remodeling chromatin states, thereby constructing multilayered regulatory pathways that govern EMT (88, 89). In the hypoxic microenvironment of NSCLC, both SOX9 (SRY-box transcription factor 9) protein levels and its lactylation are markedly increased, inducing stem-like properties in lung cancer cells and activating EMT, which in turn promotes invasion and metastasis (90, 91). Accordingly, lactylation-driven EMT is characterized by downregulation of E-cadherin and upregulation of vimentin and N-cadherin (92), a phenotype that is particularly pronounced in lung cancer cells with high AIM2 (absent in melanoma 2) expression (93).

Further studies have shown that lactylation amplifies EMT signaling through downstream gene regulation mediated by H3K9la. H3K9 lactylation upregulates the expression of laminin γ2 (LAMC2), which recruits the focal adhesion kinase/SRC (FAK/SRC) complex via integrin β1, thereby activating EMT regulators such as zinc finger E-box binding homeobox 1 (ZEB1) and Snail zinc finger transcription factor (Snail), enhancing vimentin expression, and promoting cell migration (42, 94). In addition, crosstalk between lactylation and other post-translational modifications constitutes an important layer of EMT regulation. In NSCLC, PRMT1 (protein arginine methyltransferase 1)-mediated asymmetric dimethylation of vimentin facilitates cytoskeletal remodeling, while lactylation of PRMT1 further enhances its activity, forming a positive feedback regulatory axis that promotes invasion (95).

Similarly, H3K18 lactylation strengthens both the methylation writer methyltransferase-like 3 (METTL3) and the reader YTH domain family 2 (YTHDF2), and through regulation of the METTL3–ZNF384 (zinc finger protein 384)–POLR3G (RNA polymerase III subunit G) pathway and the YTHDF2–SFRP2 (secreted frizzled-related protein 2) pathway, it coordinately modulates the expression of Snail, vimentin, E-cadherin, and SFRP2, thereby promoting lung cancer invasion (85, 96). Beyond intracellular regulation, lactylation-related signals can also spread through exosome-mediated intercellular communication. In lung adenocarcinoma, exosomes derived from tumor stem cells transfer the long non-coding RNA Mir100hg (lncRNA Mir100hg) to non-stem cancer cell populations, where it upregulates aldolase A (ALDOA) and increases H3K14 lactylation levels, activating the transcription of metastasis-related genes and consequently enhancing the metastatic potential of non-stem cells (97). This process suggests that lactylation may function as a metabolic–epigenetic signal contributing to tumor heterogeneity and the expansion of metastatic capacity (98).

Angiogenic remodeling provides the essential structural foundation for lung cancer invasion and distant dissemination. Notably, angiogenesis in lung cancer is a dynamic process regulated by both pro-angiogenic stimulators (e.g., VEGF) and inhibitory factors (e.g., solute carrier family 25 member 29, SLC25A29). Lactylation acts through the IGF1Rla/PI3K/AKT/mTOR pathway and the H3K18la/SLC25A29 pathway to simultaneously enhance pro-angiogenic signaling while relieving inhibitory constraints on angiogenesis (67, 99). In addition, the downstream H3K9la target gene AQP1 (aquaporin 1) enhances vascular permeability and synergizes with the above mechanisms to promote NSCLC metastasis (42, 100).

In fact, HIF-1α occupies a central position within these angiogenic signaling pathways. Although genes whose promoter regions undergo lactylation under hypoxic conditions are predominantly enriched in the HIF-1α signaling pathway and glycolysis/gluconeogenesis pathways, and increased HIF-1α transcription is closely correlated with lactylation levels (101, 102), HIF signaling is generally suppressed under normoxic conditions due to degradation of the HIF-α subunit. This appears paradoxical given the unique anatomical features of the lung and the Warburg effect characterized by aerobic glycolysis. One possible explanation involves the regulation of HIF-1α degradation by prolyl hydroxylases (PHDs). Lactate not only promotes HIF-1α expression through lactylation but also directly inhibits the degradative activity of PHD2, thereby inducing a state of “pseudohypoxia” that sustains HIF-1α activity. Nevertheless, current studies exhibit clear limitations and unresolved questions. On the one hand, validated lactylation-mediated regulatory effects thus far are largely indirect, and there is still no evidence that lactylation directly modifies core angiogenic factors such as VEGF or matrix metalloproteinases (MMPs). Whether the “indirectness” of these regulatory pathways affects the efficiency and specificity of angiogenic signal transduction remains to be determined. On the other hand, the amplifying effect of lactylation on the HIF-1α signaling pathway lacks quantitative support. The relative contribution of lactylation within the angiogenic regulatory axis, the precise extent to which it enhances the expression of angiogenesis-related genes (e.g., VEGF and GLUT1), and its ultimate impact on lung cancer vascular density, permeability, and metastatic capacity have yet to be clearly defined. These uncertainties constitute major bottlenecks limiting mechanistic translation and clinical application in this field.

**Figure 3:**
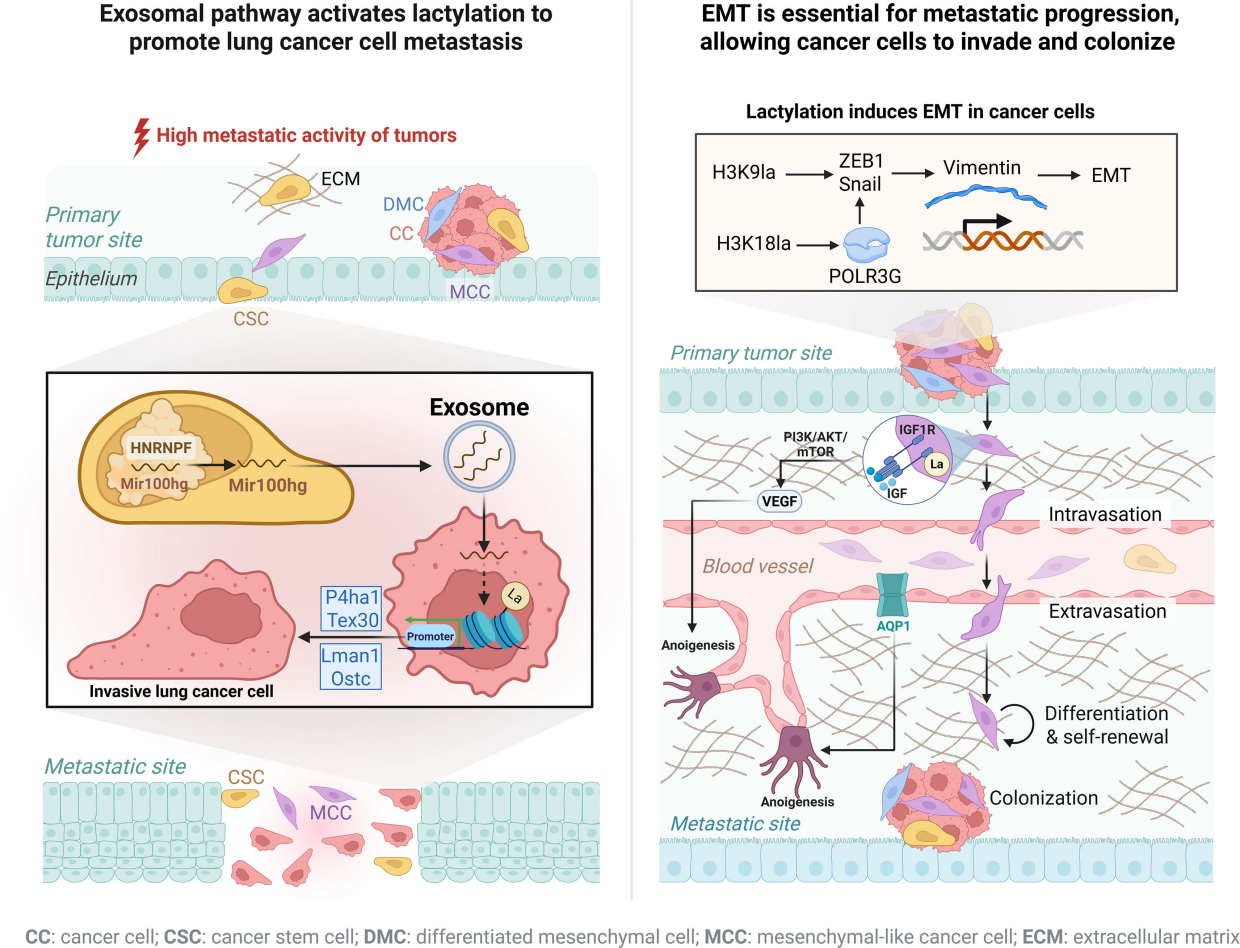
Lactylation promotes invasion and metastasis in lung cancer. Schematic depiction of lactylation-driven invasion and metastasis. Increased glycolysis and lactate accumulation enhance histone lactylation, activating transcriptional programs associated with stemness, EMT, and metastatic potential. Tumor-derived exosomes further propagate lactylation-dependent signaling in recipient cells. Lactylation activates EMT transcription factors (e.g., ZEB1, Snail), mesenchymal markers, and pro-metastatic signaling pathways, facilitating angiogenesis, dissemination, and distant colonization. Created with BioRender.

Overall, existing evidence indicates that lactylation cooperatively drives lung cancer metastasis through multilevel regulatory networks. However, its relative contribution across different lung cancer subtypes and metastatic stages has yet to be systematically elucidated, which is crucial for evaluating lactylation as a target for immunotherapy or combination treatment strategies (see **Table 2**; **Figure 3**).

**Table 2:** Lactylation-related mechanisms of lung cancer metastasis and invasion.

Type	Site	Mechanism
EMT	SOX9	SOX9/glycolysis/stemness/EMT (90)
	H3K18	H3K18la/AIM2/N-cadherin and vimentin (93)
	H3K9	H3K9/LAMC2/FAK SRC/ZEB1 and Snail/vimentin (94)
	PRMT1- K134/K145	Hypoxia/PRMT1-la/vimentin (95)
	H3K18	H3K18la/METTL3/ZNF384-m6A/POLR3G/N-cadherin and vimentin (96)
	H3K18	H3K18la/YTHDF2/SFRP2-m6A/stemness (85)
Exosome	H3K14	Mir100hg/ALDOA/H3K14la (97)
Angiogenesis	IGF1R	IGF1R/PI3K/AKT/mTOR/VEGF (67)
	H3K18/H3K14	H3K18la-H3K14la/SLC25A29/angiogenesis (99)
	H3K9	H3K9la/AQP1/angiogenesis and Vascular permeability (42)

### 3.4 Histone and non-histone lactylation cooperatively promote acquired drug resistance

**Figure 4:**
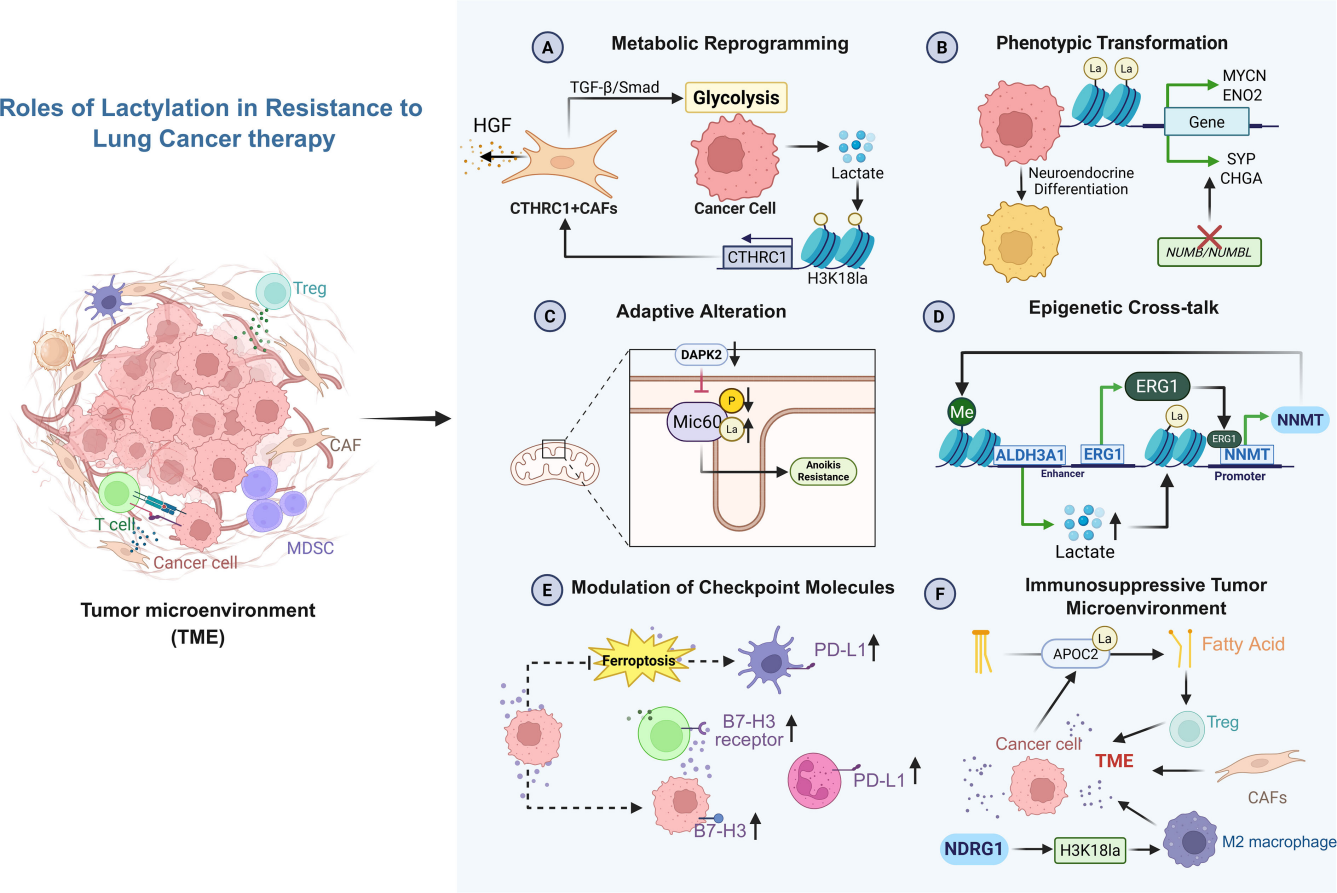
Roles of lactylation in resistance to lung cancer therapy. Overview of mechanisms by which lactylation contributes to therapeutic resistance. Lactylation promotes metabolic reprogramming, phenotypic plasticity, stress adaptation, and epigenetic remodeling, while modulating immune checkpoint expression and shaping an immunosuppressive tumor microenvironment. Collectively, these effects drive both intrinsic and extrinsic resistance to lung cancer therapies. Created with BioRender.

Therapeutic resistance in NSCLC remains the greatest barrier to achieving clinical cure. Traditionally, resistance has been attributed to four classical mechanisms: resistance-conferring mutations, activation of compensatory bypass pathways, phenotypic transitions, and metabolic reprogramming (103). However, with advances in proteomic and epigenetic technologies, recent studies increasingly suggest that these mechanisms are not independent. Instead, they are integrated into a dynamic adaptive network centered on histone and non-histone lactylation as a key epigenetic modification, providing a new perspective for understanding NSCLC drug resistance (see **Figure 4**).

In the context of targeted therapy for lung cancer, accumulating evidence consistently supports lactylation as an amplifier linking metabolic reprogramming and phenotypic drug resistance. CTHRC1^+^ cancer-associated fibroblasts (CAFs) represent a distinct subtype of CAFs characterized by lactate inducibility and elevated expression of hepatocyte growth factor (HGF). This subtype is induced by glycolysis-derived lactate through H3K18la modification at the promoter of the CTHRC1 gene. CTHRC1 in turn interacts with TGFBR2 (Transforming Growth Factor β Receptor II) via its C−terminal region, recruits TGF−β1, and activates the TGF−β/Smad3 signaling pathway, which upregulates HK2 and enhances glycolytic activity in cancer cells, thereby forming a CTHRC1/glycolysis/H3K18la positive feedback loop. Increased HGF expression subsequently maintains resistance to EGFR tyrosine kinase inhibitors (EGFR−TKIs) (34). This mechanism indicates that lactylation is not merely a downstream consequence, but rather a critical condition for stabilizing the drug-resistant microenvironment. In addition, studies on the lactate–ENO1–K89la feedback loop and the NNMT (nicotinamide N-methyltransferase)–EGR1 (early growth response 1)–ALDH3A1 (aldehyde dehydrogenase 3 family member A1) pathway further reveal dual metabolic–epigenetic positive feedback mechanisms, highlighting the high degree of self-reinforcement conferred by lactylation in maintaining resistant phenotypes (60, 62).Concurrently, H3K18la is closely associated with enhanced transcription of neuroendocrine-related genes, enabling lung adenocarcinoma cells to acquire drug resistance through lineage remodeling (104). Together, these mechanistically complementary studies support a critical role for lactylation in both the initiation and maintenance of therapeutic resistance. However, whether this role is universally applicable across all EGFR-mutant subtypes remains to be determined.

Notably, some studies have positioned lactylation at a key node of therapy pressure–driven adaptive evolution. Loss of Dapk2 (death-associated protein kinase 2) induces lactylation of Mic60 (mitochondrial contact site and cristae organizing system 60) and remodels mitochondrial cristae architecture, thereby enhancing resistance to anoikis (105), indicating that lactylation may also participate in the reprogramming of cell death pathways during the development of drug resistance. More recently, this concept has been further extended: SUMO2 and AIM2 have been identified as critical regulators of ferroptosis resistance in lung adenocarcinoma, strongly suppressing sensitivity to cisplatin treatment and responses to immunotherapy (44, 93), and playing a causal role in resistance evolution. In addition, lactylation can promote the evolutionary adaptation of tumor cells by enhancing tolerance to DNA damage and facilitating self-repair. Multiple key proteins involved in DNA non-homologous end joining (NHEJ), including FEN1 (flap endonuclease 1), XRCC5 (X-ray repair cross complementing 6), and XRCC6, have been found to be regulated by lactylation and to facilitate, at least in part, the formation of the FEN1–RAD1–RAD9A–HUS1 complex (52, 106). These findings suggest that by maintaining highly efficient DNA repair capacity and reducing lethal DNA damage induced by chemotherapy and radiotherapy, lactylation constitutes one of the important mechanisms through which tumor cells gain an evolutionary advantage under therapeutic pressure.

In immunotherapy, the role of lactylation appears to be even more context dependent. On the one hand, H3K18 lactylation promotes the expression of inhibitory immune checkpoint molecules such as B7-H3 (B7 homolog 3), providing a plausible explanation for immunotherapy failure (107). On the other hand, lactylation indirectly shapes an immunosuppressive TME through the regulation of lipid metabolism. Lactylation of APOC2 promotes the release of free fatty acids (FFAs) within the TME, inducing the accumulation of Tregs (61), while NDRG1 (N-myc downstream regulated gene 1)-driven macrophage lactylation further exacerbates the immunosuppressive state (108).Together, these studies converge on a common conclusion: lactylation does not directly block immune activation, but instead reduces the efficacy of immunotherapy by remodeling metabolic and immune microenvironments. However, systematic studies comparing the relative contribution of lactylation across different resistance mechanisms are still lacking.

Beyond the cell repair mechanisms described above, lactylation also promotes resistance to chemotherapy and radiotherapy through effects on organelle function and cell cycle regulation. Lactylation of TFEB (transcription factor EB) enhances lysosomal activity and is associated with resistance to paclitaxel and cisplatin (109, 110), while the AKR1B10 (aldo-keto reductase family 1 member B10)/glycolysis/H4K12la/CCNB1 (cyclin B1) axis contributes to acquired resistance to pemetrexed (PEM) (111). Compared with targeted therapy and immunotherapy, research in this area remains relatively fragmented and is largely confined to single-drug models, thereby limiting the integration and identification of shared mechanisms.

In summary, existing studies indicate that lactylation in NSCLC drug resistance functions more as an integrative and amplifying mechanism rather than as a single decisive triggering event. Its effects are highly dependent on treatment modality, cellular state, and the TME. This context specificity not only explains the diversity of current findings but also underscores the need for future studies to systematically delineate the hierarchical role of lactylation across different resistance pathways and to identify actionable windows for therapeutic intervention (see **Table 3**).

**Table 3:** Lactylation modification sites and mechanisms of drug resistance in lung cancer.

Treatment type	Resistant drug	Target site	Mechanism
Targeted therapy	Osimertinib	H3K18	CTHRC1/glycolysis/H3K18la (34)
	Osimertinib	H3K18	NNMT/ALDH3A1/H3K18la/NNMT (62)
	Osimertinib	ENO1	ENO1/glycolysis/ENO1-K89la (60)
	Osimertinib	Mic60	Adaptive restructuring of mitochondrial cristae (105)
	Erlotinib	H3K18	neuroendocrine differentiation (104)
Immunotherapy	anti‐PD‐1 antibody	APOC-II	promoting the release of FFAs and Tregs augmentation (61)
	anti‐PD‐1 antibody	AIM2	AIM2/STAT5B/ubiquitination (93)
	anti-PD-1 antibody	H3K18	H3K18la/B7-H3/CD8^+^T cells (107)
	anti-PD-L1 antibody	H3K18	NDRG1 Exacerbates the Suppressive State of the TME (108)
	Pembrolizumab	SUMO2-K11	SUMO2-K11 suppress ferroptosis sensitization and immunomodulation (44)
Chemotherapy	CDDP	SUMO2-K11	SUMO2-K11 suppress ferroptosis sensitization and immunomodulation (44)
	CDDP	TFEB-K91	TFEB-K91la increase lysosomal activity and autophagy flux (109, 110)
	Paclitaxel	TFEB-K91	TFEB-K91la increase lysosomal activity and autophagy flux (109, 110)
	PEM	H4K12	AKR1B10/glycolysis/H4K12la/CCNB1 (111)

## 4 Lactylation: a versatile biomarker for lung cancer diagnosis and poor prognosis

Elevated lactylation levels and their associated heterogeneity in both lung cancer cells and TME components markedly differ from those observed under normal physiological conditions. Comparative analyses revealed significantly higher lactate concentrations in lung tumor tissues than in adjacent non-tumorous regions, concomitant with substantially greater glucose uptake in lung cancer cells than in normal bronchial epithelial-like cells. Compared with that in normal lung tissue, protein lactylation is markedly elevated in lung tumor samples—particularly nuclear lactylation, which is 29.4-fold greater than that in adjacent non-cancerous tissue (112, 113). Moreover, lactylated proteins display tissue- and cell line–specific expression patterns, underscoring their potential as diagnostic biomarkers for lung cancer (5). Bioinformatic analyses based on lactylation modifications at specific sites in clinical samples have identified several lactylation-related genes closely associated with lung cancer. Following rigorous screening and validation, predictive models derived from these genes demonstrate excellent diagnostic performance, highlighting their promise as candidate molecular biomarkers (113). Additionally, lactylation-driven phenotypic states and intratumoral heterogeneity within tumor-associated stromal and immune cells—such as CAFs and Tregs—are valuable indicators for the early detection of lung cancer (34, 81, 83).

High levels of lactylation may be associated with more aggressive and metastatic lung cancer phenotypes and reduced patient survival, whereas lower levels indicate improved prognosis (114). Recently, a prognostic risk model was developed on the basis of ten lactylation-related genes significantly associated with overall survival (OS), namely, IGFBP1, CYP17A1, DKK1, KRT81, MS4A1, C11orf16, BCAN, FBN2, ANGPTL4, and SERPINB7. This model exhibited high predictive accuracy for patient OS. Subsequent *in vitro* functional studies identified KRT81 as a promising biomarker for lung cancer prognosis and disease progression (115). In a complementary approach, another study stratified lung cancers into molecular subtypes on the basis of distinct lactylation modification signatures and constructed a prognostic model using nine lactylation-related genes differentially expressed between subtypes. This work underscores the pivotal role of lactylation-associated genes in lung cancer pathogenesis and proposes that these core genes may serve as potential biomarkers of tumor stemness and poor clinical outcomes (116).

Thus far, biomarkers in thoracic oncology are mainly classified into predictive, diagnostic, and prognostic categories, encompassing diverse types such as genes, proteins, microRNAs, immune cells, and epigenetic alterations (117). Compared with established biomarkers in lung cancer, including EGFR mutations, ALK rearrangements, PD-L1 expression, and tumor mutational burden (TMB), lactylation represents a metabolism-driven, epigenetically regulated modification that integrates multiple hallmarks of tumor progression (118). While EGFR mutations and ALK rearrangements primarily serve as predictive markers for targeted therapy (119), and PD-L1 expression or TMB acts as an indicator of response to immunotherapy (120), lactylation reflects a much broader biological spectrum encompassing metabolic reprogramming, immunosuppression, and therapeutic resistance. Importantly, in contrast to static genetic alterations, lactylation is dynamically regulated by the tumor’s metabolic status and microenvironment, supporting its potential as a responsive biomarker for monitoring therapeutic efficacy or disease progression—for example, by assessing changes in lactylation scores using scoring models (121). Furthermore, lactylation-associated signatures may simultaneously capture both tumor-intrinsic and immune-related changes, potentially offering complementary predictive value beyond existing single-parameter biomarkers (122).

Although surgical biopsy remains the most widely used method for obtaining pathological tissue, it is invasive and associated with significant limitations, including high cost, procedural risk, and technical complexity (123). Recent advances in proteomics have enabled the development of robust analytical approaches—such as mass spectrometry and lactylation-specific antibody-based detection—for the comprehensive assessment of protein lactylation (124). The high resolution and sensitivity of mass spectrometry facilitate the precise identification of lactylation sites and accurate quantification of modification levels, while the availability of site-specific anti-lactyllysine antibodies markedly improves detection specificity. Together, these technologies lay a critical foundation for the clinical translation of lactylation-based diagnostics and therapeutics (125). Consequently, the systematic evaluation of global and site-specific lactylation profiles holds strong promise as a non-invasive biomarker strategy, offering valuable reference points and validation metrics for the early detection and prognostic stratification of lung cancer.

However, multiple practical challenges must be acknowledged before lactylation can be translated into clinical application. From an operational standpoint, lactylation detection relies heavily on specialized techniques such as mass spectrometry and modification-specific antibodies, which are technically demanding and time-consuming, making them unsuitable for rapid clinical decision-making. In terms of clinical feasibility, unlike conventional blood-based biomarkers such as CEA (carcinoembryonic antigen) and SCC (squamous cell carcinoma antigen), current lactylation assessments are predominantly performed on tissue samples; target molecules are present at low abundance in body fluids, and breakthroughs in high-sensitivity detection technologies are still required. With respect to diagnostic performance, existing studies lack validation in large patient cohorts to establish sensitivity and specificity. In early-stage lung cancer, where tumor burden is low, elevations in lactylation levels may be modest, increasing the risk of false-negative results. Moreover, several technical issues complicate routine detection: modification-specific antibodies may exhibit cross-reactivity; low-abundance lactylation sites are prone to interference; and the absence of standardized protocols and instrument parameters across laboratories leads to poor reproducibility. In addition, tumor heterogeneity results in temporal and spatial variability in lactylation levels within the same patient, making it difficult for a single measurement to accurately reflect disease status. Collectively, these factors substantially constrain the translation of lactylation into a routine clinical diagnostic biomarker.

## 5 From traditional metabolic intervention to novel lactylation-targeted strategies:evolution of therapy against lung cancer

### 5.1 Traditional metabolic intervention strategies targeting lactate inhibition

Although lactylation has only recently been proposed as an emerging concept, lactate itself was already recognized decades ago as being closely associated with lung cancer progression (126). With an improved understanding of lactate production mechanisms and their regulatory networks in lung cancer, metabolic interventions targeting lactate generation and transport have long been regarded as conventional strategies to suppress lung cancer progression (see **Table 4**) (127). Because lactate constitutes the biochemical basis for lactylation, inhibiting its production or transmembrane transport can, to some extent, limit lactylation levels and thereby influence tumor biological behavior.

**Table 4:** Traditional metabolic intervention strategies targeting lactate.

Drug/strategy	Molecular target	Level of intervention	Mechanism of action	Therapeutic outcome
Oxamate	LDHA	Lactate production	Inhibits conversion of pyruvate to lactate	Indirectly reduces lactylation substrate availability (128)
AT-101	LDH	Lactate production	Blocks NADH binding to LDH	Decreases lactate accumulation (128)
2-DG	HK2	Glycolysis	Competitively inhibits glucose phosphorylation	Reduces lactate production and global lactylation (129)
Fargesin	PKM2	Glycolysis regulation	Inhibits aerobic glycolysis	Decreases H3 lactylation signaling (130)
AZD3965	MCT1/2	Lactate transport	Inhibits lactate efflux	Lowers extracellular lactate (128)
Syrosingopine	MCT1/4	Lactate transport	Blocks bidirectional lactate transport	Reduces lactate-driven signaling (131)

At the level of lactate production, inhibition of key glycolytic enzymes has been shown to be an effective strategy for controlling lactate accumulation. Multiple preclinical studies have evaluated LDHA inhibitors. Among them, oxamate has demonstrated the potential to enhance the efficacy of pembrolizumab in NSCLC models, while the nonselective LDH inhibitor gossypol (AT-101), which blocks NADH binding, has advanced into phase I/II clinical trials (128). In addition, the glycolytic inhibitor 2-deoxy-D-glucose (2-DG) markedly reduces lactate production and lactylation levels by competitively inhibiting HK2 (129). Pyruvate kinase M2 (PKM2), a key regulator of aerobic glycolysis, is also a therapeutic target; its inhibitor fargesin exhibits antitumor potential by simultaneously suppressing glycolysis and H3 lactylation signaling pathways (130).

At the level of lactate transport, lactate shuttling mediated by monocarboxylate transporters (MCTs) is a key mechanism for maintaining an acidic TME and metabolic symbiosis, making MCTs another important class of therapeutic targets. The MCT inhibitors AZD3965 (an MCT1/2 inhibitor) and syrosingopine (a dual MCT1/4 inhibitor) have both demonstrated the ability to suppress lactate efflux and tumor growth in lung cancer models (128, 131). In addition, the monoclonal antibody metuzumab, which targets the MCT chaperone protein CD147 (cluster of differentiation 147), reduces MCT1/4 expression and inhibits NSCLC growth (132, 133). Notably, MCT inhibition not only affects tumor metabolism but also decreases extracellular lactate levels, thereby promoting vascular normalization and partially alleviating the immunosuppressive TME (128).

Although the strategies described above exert broad inhibitory effects at the metabolic level, their lack of specificity also introduces potential off-target risks, limiting the safety of long-term application. Moreover, resistance to glycolytic inhibitors can also emerge (134). Together, these limitations underscore the urgent need to develop novel therapeutic approaches.

### 5.2 The therapeutic potential of lactylation-based targeting

Compared with directly targeting lactate metabolism, intervening in lactylation itself or its upstream and downstream effector molecules is considered a more selective and emerging therapeutic strategy (see **Table 5**). This approach does not rely on global inhibition of glycolysis, but instead aims to precisely disrupt the pathogenic roles of lactylation signaling in tumor cells and the immune microenvironment.

**Table 5:** Lactylation-based targeted therapeutic strategies.

Therapeutic agent/strategy	Direct target	Lactylation-related mechanism	Key downstream pathway	Therapeutic outcome
Anti-APOC2 K70-lac antibody	APOC2-K70 lactylation site	Site-specific blockade of protein lactylation	Immune activation	Enhanced antitumor immune response (61)
Anti-ENO1 monoclonal antibody	ENO1	Inhibition of lactylation-associated effector function	Glycolysis–immune crosstalk	Reversal of osimertinib resistance (60)
K11-Pep	SUMO2-K11 lactylation	Peptide-based inhibition of nuclear lactylation	Ferroptosis regulation	Increased chemosensitivity and immunotherapy efficacy (44)
Shikonin	PKM2- H3K18la–AIM2 axis	Suppression of lactylation-dependent transcription	Innate immune signaling	Tumor growth inhibition (93)
GA	CTHRC1^+^ CAFs	Disruption of lactylation-driven positive feedback loop	Tumor–stroma metabolic crosstalk	Overcoming EGFR-TKI resistance (34)
NNMTi	NNMT	Modulation of metabolic–lactylation coupling	TKI sensitivity pathways	Partial reversal of TKI resistance (62)

In combination strategies involving immunotherapy, precise interventions targeting lactylation modification sites or their functional downstream effectors have shown distinct advantages. For example, a site-specific antibody against the APOC2 K70 lactylation site has been demonstrated to enhance antitumor immune responses, suggesting that lactylation sites themselves can serve as therapeutic targets (61). In parallel, combination treatment with an anti-ENO1 monoclonal antibody has restored antitumor activity in resistant models, showing promise for overcoming lactylation-associated acquired resistance to Osimertinib (60).

**Figure 5:**
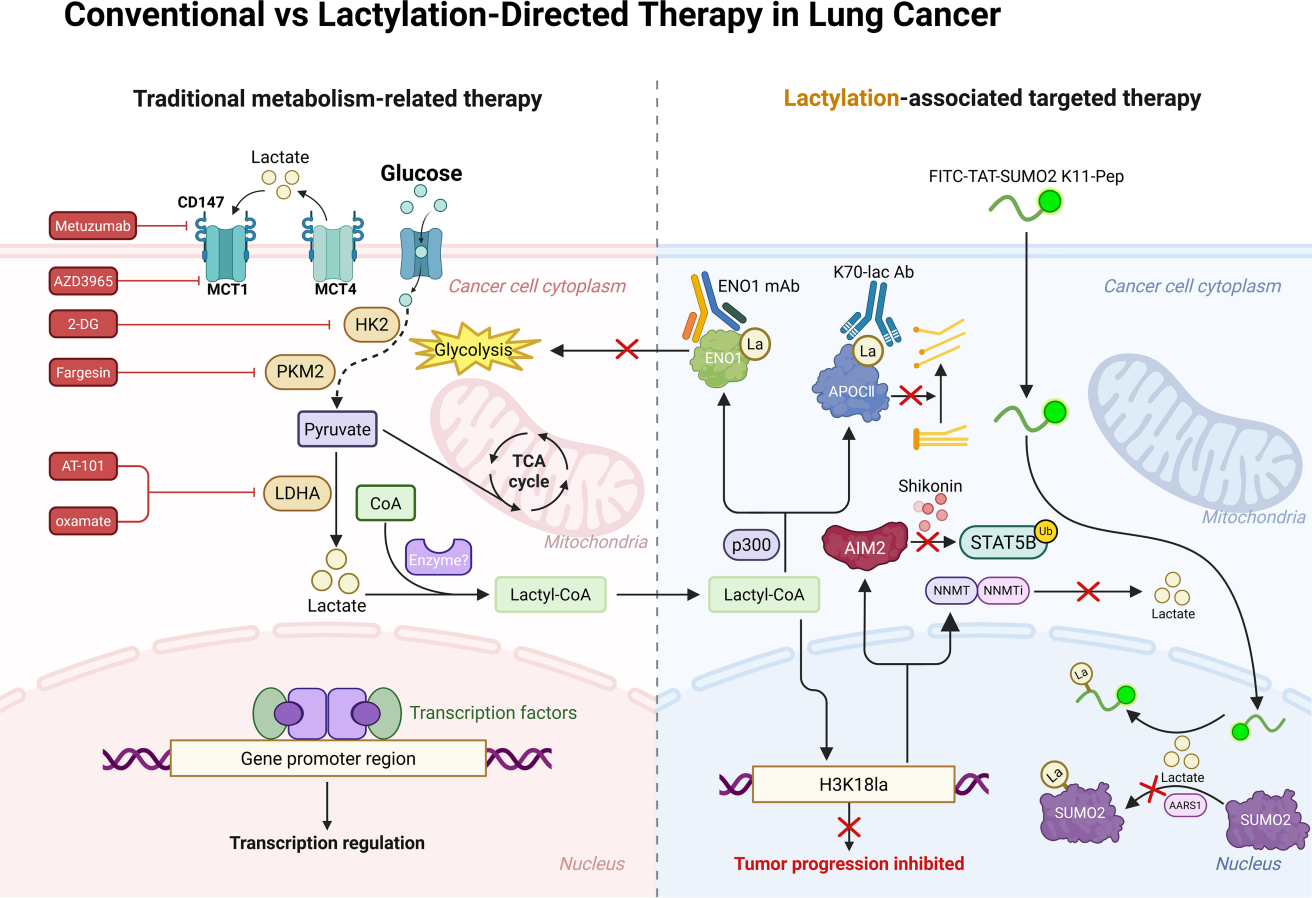
Conventional vs lactylation-directed therapy in lung cancer. Comparison between conventional metabolic therapies and lactylation-directed strategies. Traditional approaches broadly target glycolysis, lactate production, or transport, indirectly affecting tumor transcriptional programs. In contrast, lactylation-directed therapies selectively interfere with lactylation writers, lactylated residues, or lactylation-dependent signaling, offering a more precise means to disrupt lactylation-driven epigenetic regulation and tumor progression. Created with BioRender.

Beyond conventional antibodies, novel peptide inhibitors have also been developed as targeted therapeutics. A K11-Pep peptide designed against the SUMO2 K11 site exhibits high membrane permeability, enabling nuclear entry, and markedly inhibits SUMO2-K11la, thereby increasing ferroptosis sensitivity and enhancing the efficacy of chemotherapy combined with immunotherapy (44). In addition, several natural compounds have been identified to exert antitumor effects by modulating lactylation-related pathways. Shikonin, a PKM2 inhibitor, exerts anticancer effects by suppressing downstream AIM2 expression regulated by H3K18la (93). Gambogic acid (GA) shows high affinity for CTHRC1^+^ CAFs and disrupts the CTHRC1/glycolysis/H3K18la positive feedback loop, demonstrating potential for overcoming EGFR-TKI resistance (34). However, traditional herbal compounds often exhibit pleiotropic and nonspecific effects in disease treatment. Their broad pharmacological activities arise from modulation of multiple targets, which, while seemingly advantageous, complicate mechanistic dissection by obscuring the dominant pathways responsible for therapeutic effects (135). Moreover, whether multi-target–mediated biological effects contribute to adverse reactions remains to be clarified. Systematic evaluation of pharmacokinetic and toxicological properties will therefore be essential for assessing safety. Notably, the combination of a small-molecule NNMT inhibitor with osimertinib has been shown to partially reverse lactylation-driven TKI resistance phenotypes in resistant models, further supporting the feasibility of lactylation-targeted therapies (62) (see **Figure 5**).

Overall, compared with traditional metabolic inhibition strategies, lactylation-targeted therapies place greater emphasis on specific regulation and precise selection of therapeutic windows, offering clear theoretical advantages. However, their clinical translation still faces multiple deep-rooted challenges. First, the core regulatory network of lactylation has not yet been fully elucidated: Current understanding of the “writer–eraser–reader” machinery governing lactylation remains incomplete. In particular, whether lung cancer–specific lactylation-related enzymes exist is still controversial, resulting in the lack of precise and stable molecular anchors for rational drug design. Second, standardized biomarker systems and detection technologies are lacking: At present, there is no unified protocol for detecting lactylation modifications, and results obtained from different platforms, antibodies, and mass spectrometry workflows show limited comparability. Moreover, lactylation profiles exhibit pronounced heterogeneity across lung cancer subtypes, disease stages, and treatment contexts. Consequently, single biomarkers (such as H3K18la) are insufficient for reliably identifying patients who may benefit from therapy or for objectively evaluating therapeutic responses. Third, drug development and delivery face substantial technical bottlenecks: Most lactylation sites are located within the nucleus or intracellular effector molecules, requiring small-molecule drugs to overcome both cellular and nuclear membrane barriers. In contrast, peptide- or antibody-based agents often suffer from short half-lives, high immunogenicity, and poor tissue and tumor penetration, collectively constraining both therapeutic efficacy and safety margins. Fourth, evidence from clinical translational research remains severely insufficient: Existing studies are largely confined to cell lines and animal models, with a lack of large-scale, multicenter clinical cohort studies to validate efficacy and safety. In addition, the physiological roles of lactylation in normal tissues have not been fully clarified, raising concerns that long-term inhibition may lead to unpredictable metabolic or epigenetic toxicities.

Taken together, these intertwined scientific and technical challenges indicate that, although lactylation-targeted therapies demonstrate unique mechanistic advantages, a considerable distance remains before they can be successfully translated into clinical practice, established as reproducible therapeutic paradigms, and ultimately benefit patients on a broad scale.

## 6 Conclusion

Lactylation is a novel post−translational modification (PTM) that uses lactate groups as substrates and depends on the participation of multiple key enzymes. It occurs on lysine residues of both histone and non−histone proteins and plays a broad role in the progression of lung cancer. Histone lactylation directly alters gene expression to promote tumorigenesis, while non-histone lactylation drives metabolic reprogramming in tumors. These modifications often act synergistically to promote cancer development by facilitating lactylation-induced immune evasion, invasion, metastasis, and acquired drug resistance. In summary, given the extensive functional roles of lactylation in lung cancer, we systematically reviewed the enzymatic machinery involved in lactylation and its oncogenic mechanisms. We highlight the potential of lactylation as both a diagnostic and prognostic biomarker and a therapeutic target for intervention. This comprehensive analysis establishes lactylation as a critical bridge linking tumor metabolism and epigenetics in lung cancer. Therefore, targeting lactylation represents a promising strategy in cancer therapy, offering new perspectives for the development of precision treatments.

Advances in science and technology have driven breakthroughs in the study of lactylation within lung cancer; however, this field still faces several limitations. The mechanistic pathways underlying its pro-tumorigenic effects remain incompletely characterized, clinical evidence largely relies on basic experimental studies without independent validation, and translating potential therapeutic targets faces practical challenges such as insufficient specificity and unknown safety profiles. These factors constitute the primary bottlenecks preventing clinical implementation. Overall, current research is still at an exploratory stage. Progressing this field from bench to bedside will require multicenter collaboration, standardized study designs, and translational clinical research to bridge the gap between foundational discoveries and practical clinical application.
